# Acute Appendicitis in Children: Evaluation of the Diagnostic Efficacy of Ultrasonography and Computed Tomography

**DOI:** 10.7759/cureus.43860

**Published:** 2023-08-21

**Authors:** Aziz Serhat Baykara

**Affiliations:** 1 Pediatric Surgery, Health Sciences University, Eskisehir City Hospital, Eskisehir, TUR

**Keywords:** radiological diagnosis, ultrasonography, computed tomography, children, acute appendicitis

## Abstract

Objective: Acute appendicitis (AA), the most common cause of acute abdomen in childhood, can result in significant morbidity and mortality if not diagnosed and treated in a timely manner. Diagnosis of AA is more difficult in children due to the limited communication skills, in comparison to adults. The aim of this study is to evaluate the diagnostic accuracy of abdominal ultrasonography (US) and computed tomography (CT) in the diagnosis of AA in children.

Materials and methods: Pediatric patients who were operated on with the diagnosis of AA between January 2016 and December 2021 were retrospectively reviewed. Preoperative abdominal US and CT findings of the patients and postoperative pathology results were recorded.

Results: A total of 263 patients with a mean age of 11.3 years were included in the study. There were 164 (62.3%) males and 99 (37.7%) females. Histopathology revealed AA in 215 (81.7%) patients. Preoperatively, US and CT were performed in 139 (52.8%) and 137 (54.5%), respectively. Both imaging methods were applied to 13 (5.1%) patients. US had a sensitivity and specificity rate of 77.2% and 52.6%, respectively. Positive predictive value (PPV) was found to be 81.2%, whereas negative predictive value (NPV) was 46.5% for US. The diagnostic accuracy rate of US was found as 70.5%. CT had a sensitivity and specificity rate of 88.1% and 57.1%, respectively. PPV was found to be 88.8%, whereas NPV was 55.1% for CT. The diagnostic accuracy rate of CT was found as 81.8%.

Conclusion: In case of suspicion of AA, US may be the first choice because it is inexpensive and easily accessible. However, considering patient incompatibility and subjective factors in children, US may sometimes be insufficient. We think that CT should be performed as an advanced examination method in cases where US is not compatible with the patient's condition, not clinic.

## Introduction

The appendix vermiformis is a blind-ended tubular structure originating from the cecum, and the inflammation of this organ is called appendicitis. In children, acute appendicitis (AA) is one of the most common acute abdominal pathologies requiring urgent abdominal surgery. AA is the cause of 1%-4% of children with acute abdominal pain [1]. Intratubular obstruction due to fecaliths, lymphoid hyperplasia, foreign bodies, parasites, or tumors causes this clinical entity [2]. Many diseases that mimic AA, such as Meckel's diverticulitis, mesenteric lymphadenitis, gastroenteritis, upper respiratory tract infections, pneumonia, hepatitis, and familial Mediterranean fever, should be considered in the differential diagnosis [3]. Because AA has the potential to progress rapidly, early and accurate diagnosis is extremely important. In case of delays in diagnosis, serious complications such as gangrene, abscess, plastron, perforation, and generalized peritonitis may develop [4]. Morbidity and mortality rates increase in complicated cases. The risk of perforation has been reported to be 20%-70% in children with AA [3,4].

Although most patients are diagnosed with the patient's medical history and physical examination, 20%-33% of the cases present with atypical clinical symptoms [5]. The anatomical variations of the appendix, compliance problems in physical examination, and the different clinical pictures make the diagnosis more difficult in children than in adults [6]. Today, imaging methods are frequently used to achieve timely and proper diagnosis, minimize negative laparotomy rates, and prevent complicated situations [7]. The most commonly used imaging modalities are abdominal ultrasonography (US) and computed tomography (CT). Although US has high specificity and sensitivity for the diagnosis of AA, it may give false-negative results in the case of retrocecal localization [1,6]. On the other hand, CT is frequently used in cases where differential diagnosis is required or to reduce the rate of negative laparotomy. Higher accuracy rates of CT have been reported in the diagnosis of AA, compared to US [6]. In this study, the efficacy of US and CT in the diagnosis of AA in the pediatric population was evaluated and discussed in light of the literature.

This study was previously posted to the Research Square preprint server on October 26, 2022.

## Materials and methods

A total of 263 patients who were surgically operated with the pre-diagnosis of AA in the pediatric surgery clinic between January 2016 and December 2021 were retrospectively analyzed. The gender, age, abdominal US and CT findings, and surgical and histopathological data of the patients were recorded. Those reported as suspicious as results of abdominal US or CT were not accepted as AA. Appendectomy materials sent with the diagnosis of AA were examined in the pathology laboratory. The positive pathological report was based on the findings of inflammation in the tissue examined.

All procedures were in accordance with the 1964 Declaration of Helsinki and its subsequent amendments or comparable standards. Patients' informed consent was obtained to present this study. The study was approved by the local ethics committee.

Diagnostic imaging methods

Abdominal US was performed as the first imaging method in 139 patients who were admitted to the hospital with acute abdomen symptoms following the necessary clinical and laboratory examinations. While AA was diagnosed by abdominal US, the right iliac fossa was scanned with a linear transducer. Positive US results for AA were defined as non-compressible, blunt-ended tubular structures with a diameter of >6 mm, target sign on transverse examination, or presence of intraluminal hyperechoic appendicolith.

However, abdominal CT was performed on 124 patients as the first imaging method in order not to delay the diagnosis during non-working hours when the radiologists were not in the hospital. In addition, abdominal CT was performed on 13 patients whose US results were inconsistent with the patient's clinic. On CT, thickening of the appendix wall, increased streaking in the pericecal region, presence of appendicolith, and presence of free fluid in the pericecal region were considered in favor of AA [7].

Statistical analysis

Statistical analysis was performed using Statistical Package for the Social Sciences (SPSS) for Windows version 28.0.1.1 (IBM SPSS Statistics, Armonk, NY, USA). Categorical measurements were summarized as numbers and percentages, and continuous measurements as mean and standard deviation (median and minimum-maximum, where appropriate). In the study, we tried to reveal the effectiveness of radiological examinations in the diagnosis of AA by calculating the sensitivity, specificity, positive predictive value (PPV) and negative predictive value (NPV), and diagnostic accuracy rates separately between the histopathological results of the patients with abdominal US and CT. The level of statistical significance was set at p < 0.05.

## Results

Data of 263 patients with a mean age of 11.3 years (2-18) were retrospectively analyzed. There were 164 (62.3%) males and 99 (37.7%) females. All patients underwent open appendectomy with standard techniques. Postoperative histopathological assessments of the appendiceal specimens revealed AA in 215 (81.7%) patients as a definitive diagnosis. During the preoperative period, US and CT were performed in 139 (55.3%) and 137 (54.5%) patients, respectively. Both imaging methods were applied to 13 (5.1%) patients.

In the group of patients (n = 139) who were evaluated by US, 101 (72.6%) were diagnosed as AA. Preoperative US showed signs of AA in 78 cases diagnosed as AA histopathologically (true positive). However, 23 "acute appendicitis" patients had normal findings on abdominal US (false negative) (Table 1). US had a sensitivity and specificity rate of 77.2% and 52.6%, respectively. PPV was found to be 81.2%, whereas NPV was 46.5% for preoperative abdominal US. The diagnostic accuracy rate of US was found as 70.5%.

**Table 1:** Diagnostic performance of preoperative US in detecting AA (n = 139). US: ultrasonography, AA: acute appendicitis

	AA (positive)	AA (negative)	Total
US (positive)	78	18	96
US (negative)	23	20	43
Total	101	38	139

A total of 137 patients were evaluated by abdominal CT during the preoperative period. US was previously performed in 13 of these patients. Among those cases, 109 (79.5%) were diagnosed as AA at the end of the final histopathological evaluation. CT revealed AA in 96 patients (true positive), whereas 13 cases had no tomographic signs of AA (false negative) (Table 2). CT had a sensitivity and specificity rate of 88.1% and 57.1%, respectively. PPV was found to be 88.8%, whereas NPV was 55.1% for preoperative abdominal CT. The diagnostic accuracy rate of CT was found as 81.8%.

**Table 2:** Diagnostic performance of preoperative CT in detecting AA (n = 137). CT: computed tomography, AA: acute appendicitis

	AA (positive)	AA (negative)	Total
CT (positive)	96	12	108
CT (negative)	13	16	29
Total	109	28	137

When the statistical results of both diagnostic methods were compared, it was seen that CT was more advantageous than abdominal US in diagnosing childhood AA (Table 3).

**Table 3:** Comparison of statistical results between CT and US in children with AA. CT: computed tomography, US: ultrasonography, AA: acute appendicitis, PPV: positive predictive value, NPV: negative predictive value

	US	CT
Sensitivity	77.2%	88.1%
Specificity	52.6%	57.1%
PPV	81.2%	88.8%
NPV	46.5%	55.1%
Diagnostic accuracy rate	70.5%	81.8

## Discussion

AA, the most common abdominal surgical emergency in pediatric surgery, is often caused by obstruction of the appendix lumen due to fecalitis or diffuse lymphoid hyperplasia, resulting in bacterial proliferation and ischemic injury [1,4]. AA constitutes 25% of the causes of acute abdomen in adults, whereas this rate rises to 32% in children [8].

The diagnosis of AA is mainly based on careful anamnesis and detailed physical examination. However, delays in diagnosis and treatment may occur in children and confused patients due to difficulties in taking medical history and physical examination [4].

A high white blood cell count is an important laboratory finding that supports the diagnosis of AA. However, the sensitivity of leukocytosis was found to be between 19% and 60% in the previous studies [9]. Due to the low sensitivity of laboratory tests, imaging methods are commonly used especially in patients with atypical clinical symptoms. Today, the most frequently used methods are abdominal US and CT.

The purpose of imaging in the diagnosis of AA is to increase diagnostic efficiency and reduce health costs and medical risks for the patient. The use of US in the evaluation of the appendix was first described by Puylaert in 1986 [10]. Due to its advantages such as not containing radiation, being available in many centers, and being able to be used at the bedside, US has been the first choice among the imaging methods [11]. In a comprehensive and systematic review of 18 clinical studies, 77.2% sensitivity and 60% specificity rates of abdominal US were reported in the diagnosis of AA [12]. In our study, the sensitivity and specificity were calculated as 77.2% and 52.6%, respectively. In another study conducted on 238 patients, the authors reported values of 73.6% for diagnostic accuracy, 46.1% for NPV, and 73.6% for PPV [13]. In a larger study including 500 patients, the PPV, NPV, and diagnostic accuracy of abdominal US were calculated as 94.03%, 13.42%, and 70.06%, respectively [10]. In our study, the diagnostic accuracy rate of US was 70.5%, PPV was 81.2%, and NPV was 46.5%, consistent with the literature.

Since the quality and accuracy of the results for US largely depend on the skill of the person performing it, great differences can be seen in the results of various studies. Therefore, this situation can be a significant disadvantage for this examination [11]. In addition, the incompatibility of the child who has abdominal pain and whose communication skills are not yet well developed, the presence of gas-filled intestines in the right lower quadrant of the abdomen of obese patients, and the absence of high-resolution transducers are other disadvantages of the use of US [14].

Previous studies showed that abdominal CT can be more effective in making a timely and accurate diagnosis since abdominal US has a wide range of sensitivity and specificity [15-17]. Although it is recommended to perform the examination with contrast, it was stated that non-contrast CT examinations with appropriate cross sections may also be sufficient for the diagnosis [15]. In a study that evaluated the diagnostic performance of CT imaging in children with AA, a diagnostic accuracy of 95.6%, sensitivity of 97.3%, specificity of 93.7%, PPV of 89%, and NPV of 98.7% were measured [16]. In a study comparing the differences in imaging modality use and associated outcomes for AA between two pediatric hospitals in the United States and Spain, the diagnostic accuracy of abdominal CT was reported to be 94.7% and 95%, respectively [17]. In our study, the sensitivity and specificity of CT in the diagnosis of AA were 88.1% and 57.1%, respectively. In addition, PPV was 88.8%, NPV was 55.1 %, and diagnostic accuracy was 81.8%, consistent with the previous reports.

Although CT has been shown to be superior to US in the diagnosis of AA, it also has some disadvantages. For example, CT is not ubiquitous and increases health costs. Additionally, exposure to ionizing radiation, reactions due to contrast agents, and prolongation of the time until surgery are the other disadvantages [18].

Since very serious complications can develop as a result of the delay in the diagnosis of AA, surgeons generally prefer to perform appendectomy in suspected cases. Therefore, mistakes in the diagnosis of AA cause negative appendectomy [19]. Problems that may be experienced due to negative appendectomy are complications related to anesthesia, intra-abdominal adhesions, and poor quality of life due to unnecessary surgery [19]. Negative appendectomy rates in children have been reported between 1% and 40%, depending on the scope, size, and site of the studies performed in the literature [20]. In our series, our negative appendectomy rate was 14.3%.

The limitations of the study are that it is retrospective and single-centered. However, the interpretation of US and CT by different radiologists and the evaluation of appendix specimens by different pathologists have been limiting factors for diagnostic standardization.

## Conclusions

A delay in the diagnosis of AA causes serious morbidity and mortality. Therefore, one should not hesitate to use imaging methods. Since CT has higher sensitivity, specificity, positive predictive value, and diagnostic accuracy, we believe that it should be preferred, especially in suspicious cases where US is nondiagnostic or of suboptimal quality.

## References

[1] Stringer MD (2017). Acute appendicitis. J Paediatr Child Health.

[2] Almaramhy HH (2017). Acute appendicitis in young children less than 5 years: review article. Ital J Pediatr.

[3] Yilmaz M, Akbulut S, Kutluturk K, Sahin N, Arabaci E, Ara C, Yilmaz S (2013). Unusual histopathological findings in appendectomy specimens from patients with suspected acute appendicitis. World J Gastroenterol.

[4] Bolmers MD, van Rossem CC, Gorter RR, Bemelman WA, van Geloven AA, Heij HA (2018). Imaging in pediatric appendicitis is key to a low normal appendix percentage: a national audit on the outcome of appendectomy for appendicitis in children. Pediatr Surg Int.

[5] Lane MJ, Katz DS, Ross BA, Clautice-Engle TL, Mindelzun RE, Jeffrey RB Jr (1997). Unenhanced helical CT for suspected acute appendicitis. AJR Am J Roentgenol.

[6] Ceydeli A, Lavotshkin S, Yu J, Wise L (2006). When should we order a CT scan and when should we rely on the results to diagnose an acute appendicitis?. Curr Surg.

[7] Brown MA (2008). Imaging acute appendicitis. Semin Ultrasound CT MR.

[8] Pedram A, Asadian F, Roshan N (2019). Diagnostic accuracy of abdominal ultrasonography in pediatric acute appendicitis. Bull Emerg Trauma.

[9] Sack U, Biereder B, Elouahidi T, Bauer K, Keller T, Tröbs RB (2006). Diagnostic value of blood inflammatory markers for detection of acute appendicitis in children. BMC Surg.

[10] Gezer HÖ, Temiz A, Ezer S (2021). Acute appendicitis in children; assessment of diagnostic reliability of ultrasonography. Turkish J Pediatr Dis.

[11] Walter K (2021). Acute appendicitis. JAMA.

[12] Fu J, Zhou X, Chen L, Lu S (2021). Abdominal ultrasound and its diagnostic accuracy in diagnosing acute appendicitis: a meta-analysis. Front Surg.

[13] Maghrebi H, Maghraoui H, Makni A (2018). [Role of the Alvarado score in the diagnosis of acute appendicitis]. Pan Afr Med J.

[14] Rentea RM, St Peter SD (2017). Pediatric appendicitis. Surg Clin North Am.

[15] Stephen AE, Segev DL, Ryan DP, Mullins ME, Kim SH, Schnitzer JJ, Doody DP (2003). The diagnosis of acute appendicitis in a pediatric population: to CT or not to CT. J Pediatr Surg.

[16] Ahmad T, Khdair Ahmad F, Manson D (2021). Diagnostic performance of a staged pathway for imaging acute appendicitis in children. Pediatr Emerg Care.

[17] El-Gohary Y, Molina M, Chang J (2021). The use of computed tomography versus clinical acumen in diagnosing appendicitis in children: a two-institution international study. J Pediatr Surg.

[18] Musunuru S, Chen H, Rikkers LF, Weber SM (2007). Computed tomography in the diagnosis of acute appendicitis: definitive or detrimental?. J Gastrointest Surg.

[19] Bendeck SE, Nino-Murcia M, Berry GJ, Jeffrey RB Jr (2002). Imaging for suspected appendicitis: negative appendectomy and perforation rates. Radiology.

[20] Maloney C, Edelman MC, Bolognese AC, Lipskar AM, Rich BS (2019). The impact of pathological criteria on pediatric negative appendectomy rate. J Pediatr Surg.

